# Value of Clinical Reports

**Published:** 1882-03

**Authors:** 


### CLINICAL BUREAU.

### THE VALUE OF CLINICAL REPORTS.

As there are those in our school who deprecate the publishing of clinical cases, believing such reports lead to routine practice, etc., it may be well for us to state briefly their value, as the question appears to us.

First, we would say it is not fair to urge as an argument against a practice its abuse. If injury follows a practice, to make such injury an argument against that practice, it must be shown to follow from its legitimate use, not from its improper use or abuse.

There can, of course, be no value in improperly reported cases, or in fictitious cases. The case must be fully and clearly reported; so clearly that the proper remedy appears to come forward of itself, without any seeking. We have all seen such reports—reports in which the remedy, most similar to the case, seemed as apparent as the noon-day sun. And we have, it may be, wondered why we never got such *easy* cases. Not reflecting that the case appeared easy and simple to prescribe for because a master hand had drawn the picture. In the "taking" of a case lies the secret of success. If the patient be improperly examined, no amount of materia medica "thumbings" will supply the deficiency.

We believe that properly reported cases:

1. Teach how to "take" a case. Illustrating more clearly than mere instruction how our best practitioners accomplish this difficult work.

2. Illustrate the manner of prescribing; showing how minutely remedies must be individualized.

3. Indicate how to administer the *simillimum;* illustrating the various dilutions used, and teaching the comparative value of the single dose or repetition of dose.

4. Show how remedies act; some quickly, others slowly. Demonstrating the experience of our best practitioners in the important question of changing remedies or repeating doses.

5. Teach the materia medica; show the relative value of symptoms; confirm provings and bring forward new clinical hints.

